# Crystal Structures of the p21-Activated Kinases PAK4, PAK5, and PAK6 Reveal Catalytic Domain Plasticity of Active Group II PAKs

**DOI:** 10.1016/j.str.2007.01.001

**Published:** 2007-02

**Authors:** Jeyanthy Eswaran, Wen Hwa Lee, Judit É. Debreczeni, Panagis Filippakopoulos, Andrew Turnbull, Oleg Fedorov, Sean W. Deacon, Jeffrey R. Peterson, Stefan Knapp

**Affiliations:** 1 University of Oxford, Structural Genomics Consortium, Botnar Research Centre, Oxford OX3 7LD, United Kingdom; 2 Tumor Cell Biology, Fox Chase Cancer Center, 333 Cottman Avenue, Philadelphia, PA 19111, USA

**Keywords:** CELLBIO, SIGNALING

## Abstract

p21-activated kinases have been classified into two groups based on their domain architecture. Group II PAKs (PAK4–6) regulate a wide variety of cellular functions, and PAK deregulation has been linked to tumor development. Structural comparison of five high-resolution structures comprising all active, monophosphorylated group II catalytic domains revealed a surprising degree of domain plasticity, including a number of catalytically productive and nonproductive conformers. Rearrangements of helix αC, a key regulatory element of kinase function, resulted in an additional helical turn at the αC N terminus and a distortion of its C terminus, a movement hitherto unseen in protein kinases. The observed structural changes led to the formation of interactions between conserved residues that structurally link the glycine-rich loop, αC, and the activation segment and firmly anchor αC in an active conformation. Inhibitor screening identified six potent PAK inhibitors from which a tri-substituted purine inhibitor was cocrystallized with PAK4 and PAK5.

## Introduction

The control of most cellular functions relies on the spatial and temporal control of protein phosphorylation by kinases and phosphatases, and dysregulation of such signaling cascades has been linked to a large number of human diseases. The catalytic activity of protein kinases is therefore tightly regulated, and protein kinases are excellent targets for therapeutic intervention.

A molecular and mechanistic understanding of protein kinase function is essential for understanding their roles in physiology and for guiding the development of potent and selective therapeutics. All protein kinases share the same overall structure and catalytic mechanism of ATP γ-phosphate transfer. The catalytic core of protein kinases comprises two domains called the kinase lobes. The cofactor ATP binds to a cleft created by the interaction of both lobes with the hinge backbone and the glycine-rich loop that regulates ATP binding and ADP release (Aimes et al., 2000, Grant et al., 1996). Helix αC is another key regulatory element. The center of this helix contains a conserved glutamate residue that forms an ion pair with a lysine residue in active kinases. This lysine residue also coordinates the ATP α- and β-phosphates and is required for kinase activity. In addition, the αC helix often interacts with the DFG motif in the kinase activation segment, another conserved motif involved in nucleotide binding. The proximity of αC to the active site and its interactions with many conserved and essential kinase elements points to a central role in kinase regulation (Jeffrey et al., 1995, Sicheri and Kuriyan, 1997). In addition, linkage between the activation segment and αC underlies the allosteric regulation that couples substrate recognition to cofactor binding (Yamaguchi and Hendrickson, 1996).

The active state of kinases is well defined and comprises a closed lobe conformation, a well-structured activation loop suitable for recognition of the substrate, and a firmly anchored αC helix forming an ion pair with the active site lysine, enabling cofactor binding. By contrast, crystal structures of inactive kinases have revealed a large diversity of conformations, and at least one of the key regulatory elements is often displaced or disordered (Huse and Kuriyan, 2002). However, enzymatically active kinases may also crystallize in catalytically nonproductive conformations.

p21-activated protein kinases (PAKs) play central roles in a wide range of cellular processes, including regulation of cell motility, morphology, and cytoskeletal dynamics (Abo et al., 1998, Bokoch, 2003, Daub et al., 2001, Dharmawardhane et al., 1997, Kumar et al., 2006, Sells et al., 1997, Vadlamudi and Kumar, 2003). PAKs are serine/threonine protein kinases that are regulated by Rho GTPases of the Cdc42 and Rac families (Knaus et al., 1995, Manser et al., 1994, Martin et al., 1995). In humans, the PAK family comprises six members, which are classified into groups I (PAK1, -2, and -3) and II (PAK4, -5, and -6) based on their domain architecture and regulatory properties (Bokoch, 2003, Jaffer and Chernoff, 2002, Kumar et al., 2006, Zhao and Manser, 2005). Group I family members contain an N-terminal regulatory domain and a highly conserved C-terminal catalytic domain. The regulatory domain consists of a GTPase-binding domain (CRIB) and an overlapping inhibitory switch (IS) domain (Bokoch, 2003, Jaffer and Chernoff, 2002) and detailed structural and biochemical studies on PAK1 revealed the mechanism of its activation (Gizachew et al., 2000, Hoffman et al., 2000, Leeuw et al., 1998, Lei et al., 2000, Morreale et al., 2000, Thompson et al., 1998). In PAK1, residues of the kinase inhibitor (KI) segment, which acts as a pseudo substrate, bind to the cleft between the two kinase lobes. This block is released upon binding of GTP-bound Cdc42 or Rac, liberating the enzyme to undergo autoactivation by phosphorylation (Lei et al., 2000).

The mechanisms that underlie the regulation of group II PAKs is less clear since they contain no obvious autoregulatory switch domain (Jaffer and Chernoff, 2002). However, group II PAKs do contain p21-binding domains but are active in the absence of GTPases (Abo et al., 1998, Cotteret et al., 2003). Coexpression of PAK4 and Cdc42 results in translocation of PAK4 to the Golgi and the induction of filopodia, suggesting that association with GTPases plays a role in targeting group II PAKs to cellular locations (Abo et al., 1998, Dan et al., 2001). Removal of the N terminus results in an increase in kinase activity for PAK5, suggesting that group II PAKs' kinase activity might also be modulated by intra- or intermolecular interactions (Ching et al., 2003).

Comparison of five high-resolution crystal structures comprising the kinase domains of all three monophosphorylated, enzymatically active group II PAK family members revealed a number of catalytically productive and nonproductive conformers presumably representing snapshots of catalytic domain movements during catalysis. These structural rearrangements involve a sliding movement of the αC helix, adding an additional turn at the αC N terminus and a distortion of the αC C terminus. The result of this reorganization of αC is the formation of three anchor points that couple this important helix with the glycine-rich loop and the activation segment. This mechanism also distinguishes group II PAKs from the closely related group I family members in which αC moves with αA as a rigid body to bring residues important for ATP binding in close proximity to the active site. The described plasticity of group II PAKs is a prerequisite for the structure-based design of subgroup-specific inhibitors that may find applications as anticancer drugs.

## Results

### Overview of the Structures

**Figure 1:**
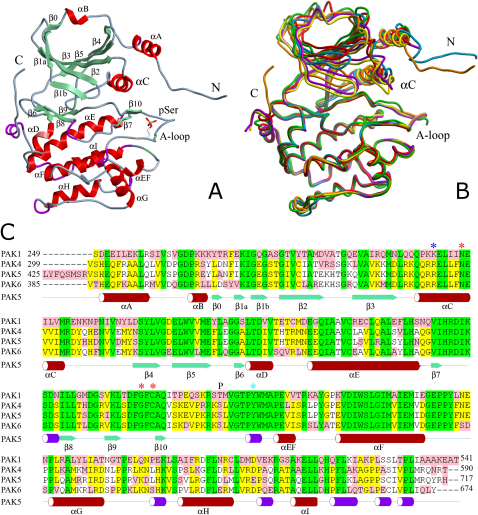
Overall Structures of Group II PAKs and Sequence Comparisons (A) Ribbon diagram showing a structural overview of PAK5. Secondary-structure elements were determined by using the program ICM Pro 3.4-8 (Molsoft LLC) and have been labeled according to a nomenclature established for PKA. The helices are shown in red, β strands are shown in green, and the 3_10_ helices are shown in magenta. The phosphorylated serine in the activation segment is shown in ball and stick representation. (B) Superimposition of all catalytic domains on Cα positions of the C-terminal lobe. Apo-PAK4 is shown in green, PAK4Etgly is shown in magenta, the PAK4 purine complex is shown in yellow, PAK5 is shown in cyan, the PAK5 purine complex is shown in orange, and apo-PAK6 is shown in red. (C) Sequence alignment of PAK4, PAK5, PAK6, and PAK1. The blue asterisk marks the Arg487 (PAK5) conserved in group II PAKs. The red asterisk indicates residues involved in the αC activation loop anchor, and the cyan asterisk indicates the putative activation segment phosphoryation site of MKK6. The autophosphorylation site is indicated by “P.” Secondary-structure elements are colored and labeled as in (A).

The structures determined in this study report the catalytic domains of all three members of the group II family (PAK4, PAK5, and PAK6). All enzymes were monophosphorylated at the activation loop positions corresponding to Ser474 in PAK4. The three catalytic domain structures comprise the typical two-domain architecture of protein kinases, with a well-ordered activation segment (Figure 1A). Constructs of the three group II PAKs included the N-terminal helices αA and αB, which are characteristic of PAK family members (Lei et al., 2000, Lei et al., 2005). All structures used were refined at high resolution to appropriate R_free_ values and had acceptable deviations form standard bond length and geometry (Table 1).

**Table 1:** Crystallographic Data and Refinement Statistics

	PAK4 (Apo)	PAK4 + Inhibitor	PAK4Etgly	PAK5	PAK6
Data Collection					

Space group	P3_2_	P4_3_2_1_2	P4_1_2_1_2	C2	P 2_1_ 2_1_ 2_1_
Cell dimensions (Å)	118.1, 118.1, 55.53	145.8, 145.8, 42.4	63.5, 63.5, 178.4	98.7, 56.6, 120.8	59.8, 66.7, 97.0
α, β, γ	90, 90, 120	90, 90, 90	90, 90, 90	90.0, 103.1, 90.0	90.0, 90.0, 90.0
Resolution (last shell)	2.3 (2.3–2.4)	2.3 (2.3–2.4)	1.6 (1.7–1.6)	1.8 (1.8–1.86)	1.60 (1.6–1.7)
Unique observations^a^	38,417 (3,826)	23,590 (2,674)	49,277 (7,986)	60,040 (5,634)	177,870 (17,251)
Completeness^a^ (%)	99.8 (100)	98.7 (92.2)	99.9 (99.5)	99.4 (94.0)	98.8 (93.6)
Redundancy^a^	5.7 (2.7)	6.7 (5.7)	7.3 (4.2)	3.7 (3.2)	3.5 (2.5)
R_merge_^a^	0.06 (0.28)	0.07 (0.41)	0.09 (0.40)	0.053 (0.39)	0.078 (0.46)
I/σI^a^	11.9 (3.5)	12.9 (4.0)	13.3 (3.6)	24.0 (3.1)	11.1 (2.1)

Refinement					

Reflections (R_free_ set)	38,386 (1,873)	19,821 (1,010)	46,301 (2,336)	56,997 (3,031)	49,551 (2,477)
R_work_/R_free_ (%)	19.2/26.8	19.9/24.8	17.4/21.5	15.4/18.5	19.7/22.2
Atoms (P/L/W)^b^	4,231/0/36	2,269/27/117	2,270/16/205	4,731/27/615	2,335/6/350
B factors (P/L/W)^b^ (Å^2^)	18.8/–/16.2	37.8/46.7/40.7	25.1/30.5/34.9	19/56/43	21/29/31
Rmsd bonds (Å)	0.007	0.012	0.008	0.010	0.016
Rmsd angles (°)	0.023	1.430	1.147	1.2	1.610
Ramachandran					
Favorable (%)	99.4	100	100	97.8	98.3
Allowed (%)	0.6	0	0	2.2	1.7

a Values in brackets represent last-resolution shell values.

b (P/L/W): protein atoms, ligand atoms, water.

The catalytic domains of the group II PAKs share about 75% sequence identity. As expected, the overall structures are similar. Cα main chain atoms of all determined structures superimpose with an rmsd of about 2 Å, and isolated lobes superimpose with an rmsd of about 0.6 Å for the C-terminal lobe and 2.1 Å for the N-terminal lobe. Superimposition on the structurally conserved C-terminal lobe of the determined group II PAK structures highlighted areas of structural diversity. The main structural differences were observed in the conformation of the glycine-rich loop, helix αC, and the N-terminal helix (Figure 1B). Group II PAKs share about 50% sequence identity with group I family members, and the superimposition of their overall structures is comparable with rmsd differences calculated between structures of group II PAKs.

Kinases are known to be extremely dynamic molecules that can adopt a large number of conformations in solution (Vogtherr et al., 2006). This plasticity is realized by a multitude of motions between and within the two kinase lobe domains that are essential for the regulation of enzymatic activity. ATP and ATP-mimetic inhibitors have been shown to stabilize closed, active conformations (Taylor et al., 2005). To shed light on this conformational plasticity of group II PAKs, we identified small-molecule inhibitors and used them to trap the kinase domains in their closed conformations. Binding of adenine-mimetic inhibitors to the active sites of PAK4 and PAK5 resulted in the expected clamping movement of the two kinase lobes and closure of the active site, as will be described below.

For this study, we defined conformations in which (i) the conserved salt bridge between the αC glutamate and the active site lysine was formed (<3 Å), (ii) the activation loop was well ordered, and (iii) the two kinase lobes were in a closed conformation as catalytically productive. In addition to the two inhibitor-trapped PAK4 and PAK5 structures, we also solved the structure of PAK4 trapped in a catalytically productive conformation in complex with ethylene glycol (PAK4Etgly). Finally, we solved the structure of noncatalytically productive apo structures of PAK4, PAK5, and PAK6. Interestingly, PAK5 crystallized with two molecules in the asymmetric unit; one molecule was found in a noncatalytically productive conformation with an unoccupied active site (apo-PAK5), and the other bound the purine inhibitor and was in a closed, catalytically productive conformation.

In the apo structure of PAK4, large regions of the kinase domain were disordered, including parts of helix αC, the glycine-rich loop, as well as part of the activation segment. Evidently, the activation segments remain quite flexible in that crystal form despite the presence of the activating phosphorylation site at residue Ser474. The significant disorder of PAK4 in this crystal form precluded detailed structural comparisons with other PAK family members. Details of all structures used in this study are provided in Table S1 (see the Supplemental Data available with this article online).

### The Activation Segment Conformation of Group II PAKs

**Figure 2:**
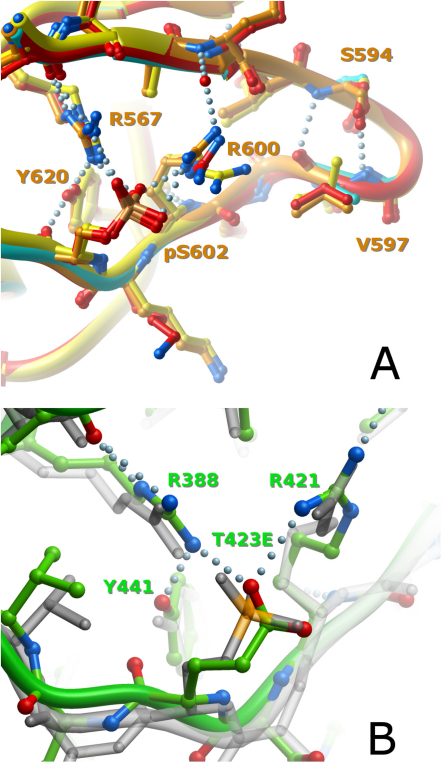
Structural Comparison of Activation Segments (A) Superimposition of the activation segments of group II PAKs showing conserved interactions: the PAK4 purine complex is shown in yellow, apo-PAK5 is shown in cyan, the PAK5 purine complex is shown in orange, and PAK6 is shown in red. The hydrogen bond network formed by the phosphoserine residue is shown, and interacting residues are labeled by using PAK5 numbering. (B) Superimposition comparing the activation segment of PAK1 (activated PAK1 mutant T423E, green) with group II PAKs (PAK5 purine complex, semitransparent); both proteins show similar activation segment conformation and interactions indicating that the glutamate residue successfully mimics the PAK1 phosphothreonine.

As with many kinases, phosphorylation of key residues in the activation segment of PAKs is required to stabilize a conformation suitable for substrate binding. The structures of the autophosphorylated group II PAKs allowed us to gain insight into how the active, catalytically productive conformation of PAKs is stabilized by this posttranslational modification. The activation loop residues important for autoactivation (Ser474 in PAK4, Ser602 in PAK5, and Ser560 in PAK6) were completely phosphorylated in all structures determined, and the presence of a stoichiometric phosphate moiety was confirmed by ESI-MS (data not shown). As expected, the activation segments of the catalytic domains were very well defined and adopted a conformation suitable for substrate binding. Interactions stabilizing this conformation were conserved in all group II PAK structures. As a representative case, the structure of PAK5 will be described in detail. In PAK5, the phosphorylated Ser602 forms a hydrogen bond network with Arg600, Arg567, Tyr620, and Phe589. This hydrogen bond network links both ends of the activation segment via the catalytic loop residue Arg567 (Figure 2A). The tip of the activation loop is further stabilized by two conserved hydrogen bonds between Ser594 and Val597. The phosphorylated Ser602 corresponds to Thr423 in PAK1, whose autophosphorylation is necessary for PAK1 activation. Although there are no structural data available on phosphorylated PAK1, a structure of an activating mutant, T423E, was recently solved (Lei et al., 2005). A comparison of this structure with the phosphorylated group II PAKs revealed that the PAK1 glutamate adopts a similar conformation and hydrogen-bonding pattern to the phosphoserine in group II PAKs, suggesting that the carboxy group mimics the phosphate moiety (Figure 2B).

### Molecular Interactions of the Trisubstituted Purine Inhibitor

The closed, active conformation represents the structural basis for the design of inhibitors targeting the active state (type I inhibitors). To capture the group II PAKs in their closed states, we sought to determine their structures bound to purine-based inhibitors. These inhibitors bind to the active site of kinases by mimicking binding of ATP and stabilize structural changes that trigger closure of the kinase lobes.

To identify small molecules that bind to PAK4, -5, and -6, the purified proteins were screened against a kinase-directed library of 605 potential low-molecular weight inhibitors by monitoring changes in protein melting temperature during thermal denaturation as previously described (Lo et al., 2004). This screening method ranks inhibitors based on an observed shift in melting temperature, which has been shown to correlate well with the binding strength and IC_50_ values (Bullock et al., 2005). Compounds that produced temperature shifts of more than 5°C were further characterized in enzyme kinetic assays. The temperature shift observed in the presence of 10 μM inhibitor corresponded well with the enzyme inhibition data (Table 2). The most potent inhibitor was the nonspecific KI staurosporine. The related molecule K252a also inhibited group II PAKs. Interestingly, three different compounds developed for the specific inhibition of cyclin-dependent kinases (cdks) and the oxoindole SU11652 were also identified as group II PAK inhibitors.

**Table 2:** Inhibitor Screening

		Tm Shift (°C)			% Activity at 10 μM		
Compound Name	Chemical Structure	PAK4	PAK5	PAK6	PAK4	PAK5	PAK6
Cdk1 Inhibitor		7.0 ± 1.5	7.1 ± 0.3	7.0 ± 0.3	56	12	23
Cdk1/2 Inhibitor III		6.5 ± 0.2	5.6 ± 0.3	5.6 ± 0.8	62	7.0	18
Purvalanol A		5.0 ± 0.3	4.5 ± 0.2	5.4 ± 0.5	20	24	48
K252a		4.5 ± 0.3	5.9 ± 0.3	8.6 ± 1.0	16	22	16
Staurosporine		13.1 ± 1.5	12.5 ± 0.3	16.6 ± 0.5	0	0	0
SU11652		6.4 ± 1.2	5.3 ± 0.2	5.3 ± 0.3	34	43	75

**Figure 3:**
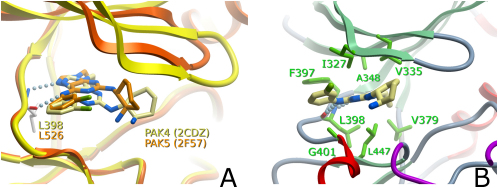
Binding of the Purine Inhibitor (A and B) Superimposition of PAK4 and PAK5 showing the (A) binding modes of the purine inhibitor and (B) interaction with active site residues in PAK4. A superimposition of the C-terminal lobes was used to generate the figure shown in (A). PAK4 is shown in yellow, and PAK5 is shown in orange.

In order to elucidate the molecular mechanisms of inhibition, we determined the structures of a 2, 6, 9-trisubstituted purine inhibitor (N-(cis-2-Aminocyclohexyl)-N-(3-chlorophenyl)-9-ethyl-9H-purine-2, 6-diamine) with PAK4 and PAK5 (Figure 3). This inhibitor was first identified as a potent inhibitor for CDKs (Dalgarno et al., 2006, Giocanti et al., 1999) and was subsequently also described as an Src inhibitor (Wang et al., 2003). In PAK4 and PAK5, the inhibitor interacted with conserved active site residues. The binding orientations of the purine scaffold in PAK4 and PAK5 differed slightly (Figure 3B); however, the mode of binding was identical to the one observed in the Src kinase complex (Dalgarno et al., 2006). As an example, the interactions of the inhibitor and the PAK4 kinase domain are described. In PAK4, and in the analogous residues in PAK5, the inhibitor forms two hydrogen bonds with the hinge backbone residue Leu398. In addition, several hydrophobic interactions with the active site residues Phe397, Ile327, Ala348, and Val335 in the N-terminal kinase lobe and Leu398, Leu447, Gly401 and Val379 in the C-terminal kinase lobe are present (Figure 3C).

### Conversion of Group II PAKs to Their Catalytically Productive Conformation Is Characterized by Three Main Motions

**Figure 4:**
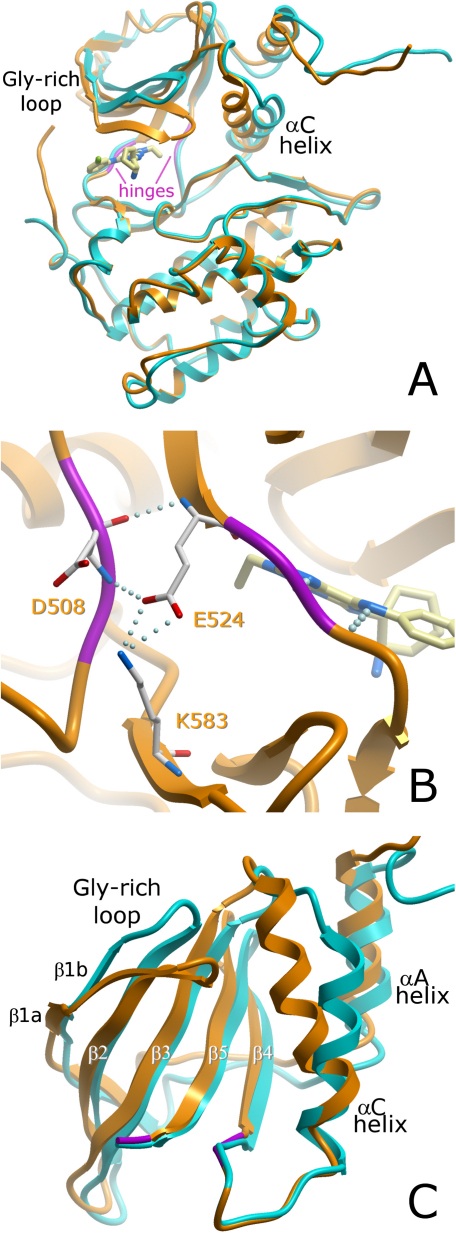
PAK5 Domain Movements (A–C) Structural rearrangements observed upon binding of the inhibitor in PAK5 (apo, cyan; purine complex, orange). (A) Superimposition with the C-terminal lobe. Hinges defined by DymDom (Hayward and Lee, 2002) are highlighted in magenta. (B) Close-up of the hinge region, rotated 180° from the view shown in (A) with the PAK5 purine complex (orange). Conserved residues forming the salt bridge linking the N- and C-terminal lobes are shown. (C) Superimposition with the core β sheet of the N-terminal lobes of apo-PAK5 (cyan) and the PAK5 purine complex highlighting the decomposed movements of the glycine-rich loop (flapping) and the αC helix (swinging).

The collective structures of the PAKs that we determined captured the enzymes in a number of states that ranged from catalytically productive to different extents of catalytically nonproductive. By examining these structures, we were able to glean a common mechanism present in group II PAKs for these structural transitions. In general, the superimposition of the large domains of the apo- and ligand-bound conformations in the various structures (Figure 4) showed a “clamping” movement that could be deconvoluted into two different smaller movements and a third swinging movement.

#### “Clamping” of the Two Kinase Lobes

The most evident change between the apo and inhibitor complexes was the expected closure of the kinase lobes induced by binding of an ATP-mimetic inhibitor, resulting in “clamping” of the ligand. The rigid body movement of the N-terminal kinase lobe toward the C-terminal lobe is similar to what is seen in PAK1 and many other kinases (Hubbard, 1997, Lei et al., 2005, Russo et al., 1996, Xu et al., 1999, Yamaguchi and Hendrickson, 1996). The detailed mechanism for the group II PAKs was best highlighted by the structure of PAK5 complexed with the purine inhibitor. In this crystal, the asymmetric unit accommodated two molecules, only one of which had the purine bound, allowing the comparison of a catalytically productive, inhibitor-bound conformation with an open, catalytically nonproductive apo structure under identical crystallization conditions. Comparison of both PAK5 conformations and the apo- and inhibitor-bound PAK4 structures identified a well-defined hinge region composed of a short stretch of the loop located C-terminal to strand β5 and the loop N-terminal to β4 (Figure 4B). It is also noteworthy that Glu524 is in the immediate vicinity of the residues involved in the hinge movement. This residue participates in a salt bridge (PAK5: Glu524–Lys583) that is conserved in both group I and II PAKs. This salt bridge is located at the interface of the C-terminal and the N-terminal kinase lobes in the open (apo) and closed (inhibitor complex) states; Glu524 belongs to the N-terminal lobe, and Lys583 is located in the C-terminal lobe, suggesting a stabilizing role in the closed conformation of PAKs.

#### Closure of the Glycine-Rich Lobe

The glycine-rich loop has been suggested to play role in fine adjustment of the ATP-binding site, and structural changes in that key regulatory element also participate in the regulation of ADP release and thereby determine the catalytic rate (Aimes et al., 2000). When the core β sheet (comprised of strands β0–β5) of the N-terminal lobe of open and closed catalytic domain conformations was superimposed, a second movement was observed in the glycine-rich loop (Figure 4C); this movement leads to further closure of the two lobes in the inhibitor-bound structure.

#### Independent Swinging of the αC Helix toward the Active Site

The N-terminal lobe helix αC represents one of the most-studied regulatory elements of kinase function. Upon activation of kinases, this helix swings toward the active site, and a conserved salt bridge between a glutamate in the αC helix and the active site lysine is formed (Canagarajah et al., 1997). A recent report suggested that in kinases like PAK1, which contain an additional helix N-terminal to the catalytic domain, the αC motion is constrained by conserved hydrophobic interactions linking the two helices, making independent αC movements impossible (Lei et al., 2005). Nevertheless, we observed an αC swinging motion independent of αA (Figure 4C) in both PAK4 and PAK5. As a consequence of this motion, the K478–E494 (PAK5 numbering) salt bridge is formed; this is one of the hallmarks of active kinases (Nolen et al., 2004). Moreover, a rotation of αC residues around the helical axis, as described for PAK1, was not observed in the six group II PAK structures, suggesting that the mechanism of the transition of group II family members from their catalytically nonproductive to their active conformation is significantly different from that of group I family members.

### Rearrangement of αC Termini Locks αC in a Catalytically Active Position and Links Key Structural Elements

**Figure 5:**
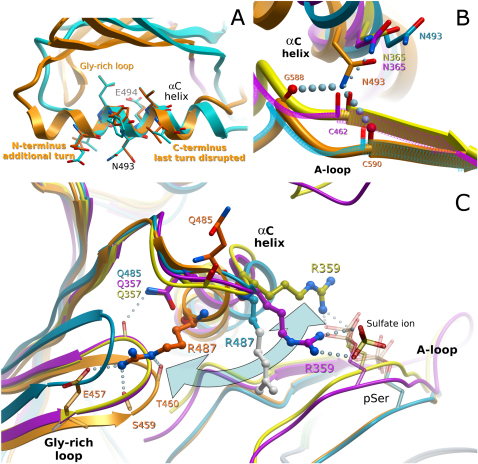
Rearrangement of Helix αC (A) Superimposition of central residues in the PAK5 αC helices showing the remodeling of the αC termini. The central residues stay in position, whereas conversion into an active state (PAK5 purine complex) results in the addition of an N-terminal α helix and disruption of the αC terminus. (B) Structural changes at the αC C terminus brings Asn493 (Asn365, PAK4) into position to hydrogen bond with the DFG glycine (Gly588) and a conserved activation segment cysteine (Cys590 and Cys462 in PAK5 and PAK4, respectively), resulting in the formation of the αC anchor point with the activation segment. In the PAK4 structures, this movement is not completed, and only one hydrogen bond is formed with Cys462. (C) Swinging movement of the conserved αC Arg487 (Arg359 in PAK4) between the glycine-rich loop and the phosphoserine activation loop residue. Upon extension of the αC helix by one turn at the N –terminus, Arg487 forms three hydrogen bonds with the glycine-rich loop, stabilizing an extremely closed conformation (PAK5 purine complex, orange). In the short αC conformation, the corresponding arginine in PAK4 interacts with the phosphoserine residue in the activation segment. This conformation also results in a partially open conformation of the glycine-rich loop stabilized by a hydrogen bond formed by the conserved Gln357. When αC swings away (as observed in apo-PAK5, cyan, or PAK6 [not shown]), the N- and C-terminal anchor points break, resulting in an open glycine-rich loop conformation. During the swinging movement, Arg487 in the PAK5 apo structure was observed in a disordered state beyond the γ carbon (indicated by white balls and sticks).

In a large number of kinase structures, the αC helix swings in and out and positions the helix close to the catalytic site in active kinases. The structures of the group II PAKs revealed a new mechanism of αC positioning that involves significant rearrangements of both helix termini (Figure 5A). In the PAK5 inhibitor complex, the last turn of the αC helix is distorted and becomes a loop region, thus adding length to the αC–β4 loop. In contrast, the N terminus of αC gains an additional turn with the rearrangement of the βC–αC loop. This resulted in the “shifting” of the αC lengthwise toward its N terminus, yet the spatial position of the central residues remained unaltered along the main axis of the helix. Similar rearrangements of helix αC were also observed when the PAK4 inhibitor complex was compared with PAK4Etgly, suggesting that this novel, to our knowledge, mechanism is conserved in group II PAKs (not shown).

A consequence of the shift in the αC is the formation of new interactions that anchor this helix in its active conformation and link αC with the glycine-rich loop and the activation segment. Rearrangement of the αC C terminus results in the formation of an anchor point that links αC with the activation segment (Figure 5B). Disruption of the αC C terminus moves Asn493 (PAK5) and Asn365 (PAK4) into position to form a hydrogen bond with the conserved activation segment residue Cys590 (Cys462 in PAK4) and with the DFG motive Gly588 (PAK5).

The shift in register of the αC helix toward the N terminus results in the formation of hydrogen bonds that position the glycine-rich loop in a closed conformation competent for interactions with phosphate moieties of the ATP cofactor. In the PAK5 purine complex, the αC N-terminal extension leads to the formation of two hydrogen bonds, between the conserved αC Arg487 and main chain residues of the glycine-rich loop residues Ser459 and Gly458, respectively, as well as the formation of a salt bridge by the carboxyl group of Glu457 (Figure 5C). In the catalytically nonproductive PAK5 apo structure, Arg487 is disordered after the γ carbon. However, the orientation of the defined portion of that side chain shows that Arg487 moves toward the activation loop. We believe that the endpoint of that movement has been captured in the two closely related PAK4 structures. In the PAK4EtGly structure, the corresponding arginine (Arg359) forms hydrogen bonds with the phosphate moiety of the phosphorylated activation loop residue S474; in the PAK4 purine complex, Arg359 forms an indirect interaction with that residue via a sulfate ion present in the crystallization solution. In this state, a more open conformation of the glycine-rich loop is stabilized by a hydrogen bond between the αC residue Gln357 and the main chain oxygen of Thr332. It is interesting to note that at this stage the “growing” αC helix is already stabilized by the typical hydrogen bond connectivity between Gln357 and Asp353 main chain atoms in helices 1–4, whereas the backbone is still in loop conformation.

Thus, structural comparison of the six high-resolution crystal structures of group II PAKs identified structural rearrangements in the transition from a catalytically nonproductive, open state to an active, closed state that are significantly different from motions described for the group I member PAK1 (Lei et al., 2005), and, to our knowledge, it identified structural rearrangements never reported before in protein kinases. The described structural changes link key regulatory elements such as the glycine-rich loop, αC, and the activation segment, giving structural insight into how group II PAKs control catalysis and recruitment of the cofactor ATP as well as the release of ADP.

## Discussion

In this study, we used inhibitors and different crystal forms to trap PAKs' catalytic domains in a number of conformations. The body of structural information allowed us to describe structural rearrangements that occur during the transition from catalytically nonproductive, open states to an active, catalytically productive, closed state of group II PAK enzymes.

PAKs and many other kinases are regulated by phosphorylation of key residues in their activation segment (Nolen et al., 2004). Phosphorylation at these sites shifts the equilibrium between the multiple catalytically nonproductive states to the active state of the enzyme. Our study, like any crystallographic analysis, assumes that conformational changes identified by structural comparisons resemble conformational changes occurring in solution. In this study, we confirmed domain movements and interactions in a number of different crystal forms, and the observed conservation of interactions within this closely related family strongly suggests that the observed interactions are not simply a consequence of crystallization or crystal packing. Recently, an NMR study confirmed a high degree of mobility in and around the ATP-binding site of the kinase catalytic domain, suggesting large-scale conformational changes even after binding of high-affinity inhibitors (Vogtherr et al., 2006).

Unlike the group I PAKS, the group II enzymes lack obvious autoinhibitiory domains. However, group II PAKs still interact with GTPases; these interactions target the kinases to certain cellular locations, but they have no influence on enzymatic activity (Abo et al., 1998, Dan et al., 2001). The lack of IS domains suggests that group II PAKs are constitutively active enzymes and rapidly autoactivate by activation segment phosphorylation (Cotteret and Chernoff, 2006). However, removal of the N terminus also results in an increase in kinase activity for PAK5, suggesting that enzymatic activity may be modulated by interactions with the N terminus (Ching et al., 2003). To our knowledge, the structures discussed here represent the first active PAK kinase structures activated by phosphorylation rather than by a phosphomimetic mutant. However, a comparison of the PAK1 activation mutant structure (Lei et al., 2005) with the phosphorylated group II PAKs showed that the PAK1 glutamate carboxy group mimics the phosphate moiety well.

Studies on cellular stress-response pathways suggested that PAK5 and PAK6 are linked to this signaling cascade by an activating phosphorylation on the consensus MAP kinase kinase 6 (MKK6) site (Thr-Pro-Tyr) in the activation loop (Kaur et al., 2005, Maroni et al., 2000). The structures of group II PAKs showed that this consensus motif is embedded in a deep cleft and is not accessible. Thus, phosphorylation at this site would require significant rearrangement of the neighboring residues

**Figure 6:**
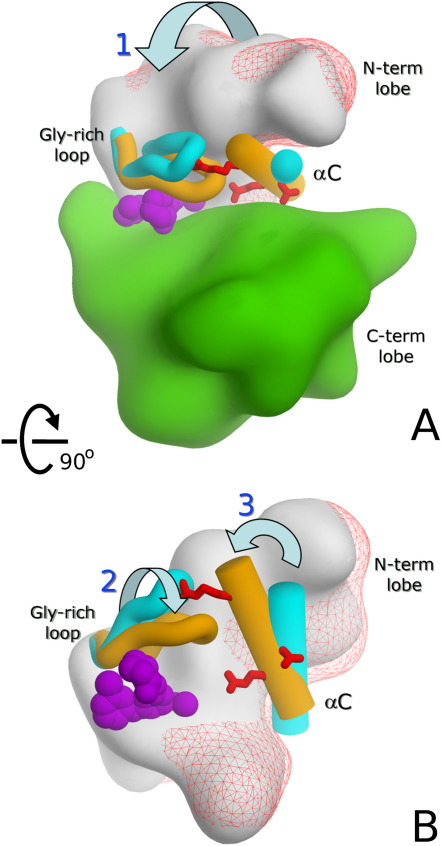
Model Showing Conformational Changes in Group II PAKs (A and B) The N-terminal lobe is represented as red wire-frame (apo) and white surface (closed conformation). Secondary-structure elements involved in the domain movements are shown and colored in cyan (apo) and orange (closed conformation). Red sticks indicate positions of the three anchor points (see main text), and magenta CPK spheres depict the purine inhibitor. The clamping (movement 1) is shown in the (A) overview, while the detailed view in (B) depicts the closure of the cofactor binding site (movement 2) and the repositioning of αC (movement 3).

Comparison of the six high-resolution structures revealed the domain movements conserved in this subfamily, suggesting a model (Figure 6) for the transition of the inactive states of the enzyme to a conformation competent for catalysis. The deconvolution of the allosteric clamping of the two lobes into smaller movements was critical to clarify the seemingly confusing range of conformations adopted by the N-terminal lobe (Hayward, 2004, Hayward and Lee, 2002). When we examined the clamping of the cofactor through the closure of the two lobes, it was evident that residues involved in the hinging of the lobes are not responsible for the binding of ATP. The distinction of the residues binding the cofactor from residues involved in the hinge movement can be rationalized as a necessity to maintain the cofactor bound during the movement without compromising the geometry and interactions provided by the cofactor-binding site. The salt bridging of the N-terminal lobe through Glu524–Lys583 (PAK5 numbering) located on the C-terminal lobe suggests an anchoring role for theses residues during the hinge movement (Figure 6A).

The second movement involving the glycine-rich loop seems to serve for further adaptation of the N-terminal lobe to the bound cofactor or ATP-mimetic inhibitor (Figure 6B). A possible biological function for this loop might be to act as a molecular sensor. Accordingly, superimposition of the active conformation of PAK5 with the TAO2 kinase structure (Zhou et al., 2004) bound to ATP showed that the position occupied by the tip of the glycine-rich loop is in close proximity to the γ-phosphate position of ATP (i.e., the phosphate that is transferred to the substrate), whereas the bulk of the glycine-rich loop is within hydrogen-bonding distances to β- and α-phosphates (Figure S1, see the Supplemental Data available with this article online). This arrangement allows the glycine-rich loop to either be employed as a steric constraint to force the γ-phosphate into a position favorable for catalysis or to serve in the trapping of ADP after hydrolysis, controlling the initiation of the next catalytic cycle. This hypothesis agrees with previous reports on enzyme kinetic studies on protein kinase A (PKA) that suggest that the final step of ADP release is rate limiting (Adams, 2003, Aimes et al., 2000, Grant et al., 1996). We speculate that the conformation of the glycine-rich loop and the observed interactions play a role in regulating the release of ADP and the rebinding of ATP, which marks the beginning of a new catalytic cycle (Lu et al., 2005). The shifting of the αC helix links this important structural element to the glycine-rich loop. It is therefore likely that the formed hydrogen bond network as well as steric constraints created by the additional helix turn regulate kinase activity.

Our structural analysis suggests that the correct positioning of αC helix is the most critical of the precatalytic events. The six structures compared identified three anchor points that lock helix αC in the fully active conformation. Two of the anchor points form after the shifting of helix αC, adding a new turn at its N terminus. This shifting movement is not likely to be present in group I PAKs that contain a proline residue N-terminal to αC, not allowing further N-terminal extension. As a consequence, group I PAKs rotate αC around the helical axis rather than shifting it (Lei et al., 2005).

The most striking consequence of the described interactions is that all key regulatory elements in group II PAKs are linked. The N-terminal helix αC expansion brings the conserved group II Arg487 (PAK5) into position for tight interaction with the glycine-rich loop, stabilizing closure; however, in the inactive state, this residue interacts with the phosphate moiety of the activation loop phosphoserine, which links ATP and substrate binding. Coupling of activation segment phosphorylation and positioning of αC has also been observed in the tyrosine kinase IRK, where the conformation of the unphosphorylated activation loop is also correlated with inactive orientations of helix αC (Hubbard et al., 1994).

Understanding the plasticity of the enzymes is also a prerequisite for structure-based inhibitor design. Targeting inactive conformations of kinases led to the development of highly selective inhibitors. The anticancer drug Gleevec, for instance, selectively binds to an inactive conformation of Abl kinase (Schindler et al., 2000). In their inactive states, the closely related Src family members differ in their activation loop conformation. Consequently, Src kinases do not bind Gleevec despite close sequence similarity. As demonstrated in this study, the mechanism of αC positioning in the active conformation of group II PAKs differs significantly from that of group I family members. This activation process also influences the conformation of the glycine-rich loop, which has been shown to regulate binding of ATP inhibitors and may even block access to the active site completely in some kinases (Shewchuk et al., 2000). Thus, the structural information presented here describing the plasticity of group II PAKs and the differences to group I family members could be exploited to develop inhibitors that are selective for this subfamily of enzymes.

In this study, we also identified several inhibitor scaffolds by using a fluorescence-based temperature-shift screening assay. The 2, 6, 9-trisubstituted purine inhibitor (CGP74514A) has been described as a potent and cell-permeable inhibitor of Cdk1/cyclin B (Giocanti et al., 1999, Wang et al., 2003). It has been shown to cause mitochondrial damage and apoptosis in several human leukemia cell lines and, as expected, leads to G_2_M cell cycle arrest at lower concentrations (Aleem et al., 2005, Dai et al., 2002, Yu et al., 2003). The identified inhibitor scaffolds, which inhibit PAK kinases, provide valuable starting points for further development of more potent and selective inhibitors.

The group II PAKs have been linked to many cellular processes important for tumorigenesis (Bokoch, 2003, Kumar et al., 2006, Vadlamudi and Kumar, 2003), including cell transformation, anchorage-independent cell growth, and apoptosis, and PAK4 is overexpressed in many cancer types (Callow et al., 2002). In addition, PAK6 has been reported to be responsible for regulation of androgen receptor signaling in prostate cancer (Schrantz et al., 2004, Wang et al., 2005). The described high-resolution structures can be utilized to optimize these compounds further to develop selective and more potent inhibitors of these kinases for the treatment of human diseases.

## Experimental Procedures

### Cloning

Catalytic domain residues were amplified from cDNA provided by the mammalian gene collection (MGC). PAK4 (residues 300–591) was cloned into the SmaI site of pGEX-6P2; PAK5 (residues 425–715) and PAK6 (383–674) were cloned into the vector pNIC28-Bsa4 by ligation-independent cloning (Stols et al., 2002). The vector includes a TEV-cleavable (^∗^), N-terminal His_6_ tag (MHHHHHHSSGVDLGTENLYFQ^∗^SM).

### Expression and Purification

Transformed BL21(DE3) cells were grown in Luria-Bertani medium containing 100 μg/ml ampicillin (PAK4) or kanamycin (PAK5, PAK6). Protein expression was induced at an OD_600_ of 0.8 by using 1 mM isopropyl-thio-galactopyranoside (IPTG) at 18°C for 12 hr. Cells expressing His_6_-tagged PAKs were lysed in 50 mM HEPES (pH 7.5), 500 mM NaCl, 1 mM PMSF, and 0.5 mM TCEP by using an EmulsiFlex high-pressure homogenizer. After centrifugation, the supernatant was loaded onto a Nickel-Sepharose column equilibrated in 30 ml binding buffer (50 mM HEPES [pH 7.5], 500 mM NaCl, 5 mM imidazole, 0.5 mM TCEP, 5% glycerol). The column was washed three times with 10 ml wash buffer (loading buffer with 30 mM imidazole). Proteins were eluted by an imidazole step gradient and were applied to a Superdex 200 16/60 gel-filtration column equilibrated in 50 mM Tris (pH 8), 150 mM NaCl, 5 mM DTT.

Cells expressing GST-PAK4 were lysed in 50 mM Tris-HCl (pH 8), 150 mM NaCl, 5 mM DTT. The supernatant was bound to glutathione Sepharose, and the resin was washed with loading buffer and incubated with GST-tagged PreScission protease (∼50 μg per mg) for 12 hr. PAK4 protein was eluted and further purified by gel filtration. The purified proteins were homogeneous, as assessed by SDS-PAGE and electrospray mass spectrometry.

### Crystallization

Crystallization was performed by using sitting drops, which mixed protein (8–10 mg/ml) and well solutions in 2:1, 1:1, and 1:2 ratios. PAK4 hexagonal apo crystals (2BVA) were obtained with a well solution containing 1.5 M NaCl and 10% (v/v) ethanol. Tetragonal crystals of PAK4 were obtained with 0.20 M tripotassium citrate, 0.1M BTProp (pH 6.5), 20.0% PEG 3350, 10.0% EtGly. PAK6 was crystallized by using 1.60 M magnesium sulfate, 0.1 M MES (pH 6.5). The PAK4 inhibitor complex crystals were obtained by using 1.2 M ammonium sulfate, 15% PEG 200, and 100 mM Tris (pH 8.0). PAK5 protein apo and complex were crystallized by using 0.20 M Na/KPO_4_, 0.1 M Bis Tris propane (pH 7.5), 20.0% PEG 3350, and 10.0% etylene glycole.

### Data Collection and Processing

The data were collected at the Swiss Light Source (SLS). Data collection was performed on flash-frozen crystals at 100K, and 15% glycerol was used as cryoprotectant. Images were indexed and integrated by using MOSFLM and were scaled with SCALA (Evans, 1993), part of the CCP4 suite of programs. Data collection statistics and cell parameters are listed in Table 1.

### Structure Solution and Refinement

PAK4 and PAK6 structures were solved with molecular replacement by using Phaser (Storoni et al., 2004) and the human PAK1 (PDB ID: 1F3M) as a search model, whereas PAK6 was used as a model to solve PAK5. Iterative rounds of rigid-body refinement and restrained refinement with TLS, against maximum likelihood targets, were interspersed by manual rebuilding of the model by using Coot (Emsley and Cowtan, 2004) and Xfit/XtalView (McRee, 1999).

### Thermal Stability Measurements

Thermal melting experiments were carried out with an Mx3005p real-time PCR machine (Stratagene). Proteins were buffered in 10 mM HEPES (pH 7.5), 150 mM NaCl and were assayed in a 96-well plate at a final concentration of 2 μM in a 20 μl volume. Inhibitors were added at a final concentration of 10 μM. SYPRO-Orange (Molecular Probes) was added as a fluorescence probe at a dilution of 1 in 1000. Excitation and emission filters were set to 465 nm and 590 nm, respectively. The temperature was raised with a step of 1°C per minute, and fluorescence readings were taken at each interval. The temperature dependence of the fluorescence was approximated by the equation

(1) $$y\left(T\right)=y_{F}+\frac{y_{U}-y_{F}}{1+e^{\Delta uG_{\left(T\right)}/RT}},$$

where ΔuG is the difference in unfolding free energy between the folded and unfolded state, R is the gas constant, and y_F_ and y_U_ are the fluorescence intensity of the probe in the presence of completely folded and unfolded protein, respectively (Matulis et al., 2005). The baselines of the denatured and native state were approximated by a linear fit. The observed temperature shifts, Δ*T*_m_^obs^, for each inhibitor were recorded as the difference between the transition midpoints of sample and reference wells containing protein without inhibitor and were determined by nonliner least-squares fit.

### Enzymatic Assays

In vitro kinase assays were carried out by using 100 nM PAK and 8 μM substrate (MBP) in phospho-buffer (50 mM HEPES [pH 7.5], 12.5 mM NaCl, 0.625 mM MgCl, 0.625 mM MnCl). This mixture was added to dilute compounds such that the final inhibitor concentration was 10 μM at 1% DMSO. Reactions were started by the addition of 20 μM ATP mixed with ^32^P-ATP, carried out for 10 min at 30°C, and they were stopped by boiling at 95°C. Samples were spotted onto P81 paper (Whatman), washed on 0.1% phosphoric acid, and analyzed by scintillation counting. PAK activity was expressed as the percent activity compared to control (1% DMSO) reactions.

### Compounds

Staurosporine was purchased from LC laboratories. All other compounds were purchased from EMD Biosciences.

## Accession Numbers

The crystal structures reported in this paper have been deposited in the Protein Data Bank (http://www.pdb.org) with accession codes 2BVA, 2JO1, 2CDZ, 2F57, and 2C30.

## Supplemental Data

Document S1. One Figure and One Table
