# Supplementary material for: Crystal Structures of the p21-Activated Kinases PAK4, PAK5, and PAK6 Reveal Catalytic Domain Plasticity of Active Group II PAKs
Source: Structure. 2007 Feb;15(2):201–13. doi: 10.1016/j.str.2007.01.001 (PMC1885963; doi:10.1016/j.str.2007.01.001)
Supplement: Document S1. One Figure and One Table [file mmc1.pdf]

Structure ***15***

Supplemental Data

Crystal Structures of the p21-Activated Kinases PAK4, PAK5, and PAK6 Reveal Catalytic Domain Plasticity of Active Group II PAKs

Jeyanthy Eswaran, Wen Hwa Lee, Judit É. Debreczeni, Panagis Filippakopoulos, Andrew Turnbull, Oleg Fedorov, Sean W. Deacon, Jeffrey R. Peterson, and Stefan Knapp

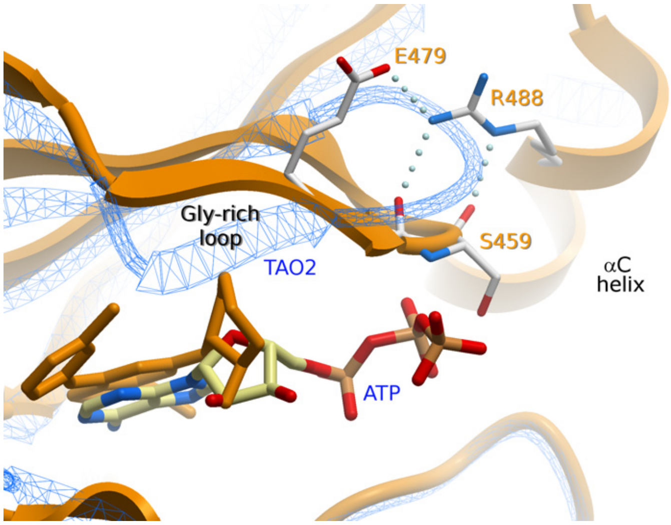

Figure S1. Glycine Rich Loop as a Potential Sensor for the Presence of Cofactor ATP γ-Phosphate

Structures of PAK5 (orange) in complex with the purine inhibitor and TAO2 (blue wireframe) in complex with ATP were superimposed using C-terminal lobe C-alpha positions.

Table S1. Comparison of Group II PAK Conformation

Structure (PDB)	Ligand in active site		Position and linkage of key elements			State
			Latch I	Latch II	Latch III	
		Distance E-K	Glycine rich loop conformation	H-bond between αC-helix and Glycine- rich loop	H- bond between αC- helix and A-loop	
PAK4 Chain A (**2BVA**)	none	4.0Å	disordered	not applicable	N365-C462	Catalytically non-productive
PAK4 Chain A (**2BVA**)	none	7.3Å	disordered	not applicable	none	Catalytically non-productive
PAK4 EtGly (**2JO1**)	3 EtGly	2.6Å	intermediate	Q357 to T332, D353	none<br>R359 to A loop phosphoserine	Catalytically productive but missing latch II
PAK4 Inhibitor (**2CDZ**)	Purine	2.5Å	intermediate	Q357 to T332, D353	N365-C462<br>indirect interaction R359 to A-loop phosphoserine.	Catalytically productive
PAK5 Chain A (**2F57**)	none	5.3Å	open	none	none	Catalytically non-productive
PAK5 Chain B (**2F57**)	Purine	2.7Å	closed	R487 to S459. G458, E457	N493 to C590 and G508	Catalytically productive
PAK6 (**2C30**)	none	4.7Å	open	Q443 to T418	none	Catalytically non-productive
PAK1 Activating Mutant (**1YHV**)	none	3.4Å	intermediate	Q306 to A280	none	Catalytically non-productive
